# Anemia, malnutrition and their correlations with socio-demographic characteristics and feeding practices among infants aged 0–18 months in rural areas of Shaanxi province in northwestern China: a cross-sectional study

**DOI:** 10.1186/1471-2458-12-1127

**Published:** 2012-12-29

**Authors:** Wenfang Yang, Xu Li, Ying Li, Shuiping Zhang, Liming Liu, Xiang Wang, Weimin Li

**Affiliations:** 1 Maternal and Child Health Center, The First Affiliated Hospital of Medical College in Xi’an Jiaotong University, No. 277, Yanta West Road, Xi’an City, Shaanxi Province 710061, P.R. China; 2 Family Planning Service Center of Xi’an, Xi’an City, Shaanxi Province 710003, P.R. China; 3 Xi’an Municipal Maternal and Child Health Hospital, Xi’an City, Shaanxi Province 710002, P.R. China

**Keywords:** Infants, Malnutrition, Anemia, Micronutrient deficiency, Cross-sectional study

## Abstract

**Background:**

The first 18 months of life are the most important for long-term childhood well-being. Anemia and malnutrition occurring in this key period have serious implications for individuals and societies, especially in rural areas in developing country. We conducted a cross-sectional study as the baseline survey to provide data for developing a policy-based approach to controlling infant anemia and malnutrition in rural areas of Shaanxi province in northwestern China.

**Methods:**

We randomly sampled 336 infants aged 0–18 months in 28 rural villages from 2 counties of Shaanxi province. Anthropometric measurements and household interviews were carried out by well-trained researchers. The hemoglobin concentration was measured for 336 infants and serum concentrations of iron, zinc, and retinol (vitamin A) were measured for a stratified subsample of 55 infants. Anemia was defined using World Health Organization (WHO) standards combined with the Chinese standard for infants <6 months old. Logistic regression modeling was used to estimate the odds ratios (ORs) and 95% confidence intervals (CIs) for anemia with non-anemic group as a reference.

**Results:**

We found that 35.12% of infants in rural Shaanxi suffered from anemia, and the malnutrition prevalence rates were 32.14% for underweight, 39.58% for stunting, and 11.31% for wasting. Anemia was significantly associated with malnutrition (underweight, OR: 2.42, 95%CI: 1.50-3.88; stunting, OR: 1.65, 95%CI: 1.05-2.61; wasting, OR: 2.89, 95%CI: 1.45-5.76). Low birth weight, more siblings, less maternal education, low family income, crowded living conditions, and inappropriate complementary food introduction significantly increased the risk for infant anemia. Serum concentrations of iron, zinc, and retinol (vitamin A) were significantly lower in anemic infants compared with non-anemic infants.

**Conclusions:**

Specific socio-demographic characteristics and feeding patterns were highly associated with infant anemia in rural areas of Shaanxi province. Health education focusing on feeding practices and nutrition education could be a practical strategy for preventing anemia and malnutrition in young children.

## Background

Child anemia and malnutrition have both short- and long-term adverse consequences that have serious implications for individuals and societies. It continues to be a major health burden in developing countries and is a substantial contributor to childhood morbidity and mortality [1-3]. In the past 30 years, a series of economic reforms have greatly improved general living standards and health levels in China, and mortality rates of children under 5 years old have decreased significantly [4-7].

However, certain areas of the country continue to have high malnutrition rates. In northwestern China, child mortality persists at 60.8/1000 live births in children under 5 years old and 49.2/1000 live births in infants as of 2000, and malnutrition remains one of the main causes of childhood mortality [8]. Previous studies have reported that these issues are more serious in northwestern China where economic levels are lower and geographical environments are worse, especially in large rural areas [9].

The first 18 months of life are the most important for long-term childhood well-being. It is known that insufficient food intake in this period is common, and inadequate breast-feeding or complementary feeding is responsible for growth stunting and infant morbidity, including nutritional anemia in millions of children around the world [10].

We conducted a cross-sectional study as the baseline survey to provide data to develop a policy-based approach for controlling infant anemia and malnutrition in rural northwestern China. The objectives of the study included: (1) Measure the prevalence of anemia and malnutrition among infants less than 18 months old in Shaanxi province of northwestern China; (2) Detect the socio-demographic, feeding practice risk factors and nutritional factors for these problems.

## Methods

### Study population and design

A cross-sectional study on malnutrition and anemia prevalence among infants aged 0–18 months was conducted in 2 rural counties in central Shaanxi province in northwestern China. A sample of 280 infants was estimated to be able to detect a 25% anemia rate and 8% acceptable variability, assuming *α* = 0.05, *β* = 0.20, and a design effect = 2.5. The sample size was expanded to 336 infants to account for a 20% non-response rate. We sampled 336 infants from 5 stratified age groups of 0–3 months, 4–6 months, 7–9 months, 10–12 months and 13–18 months. 11 infants in each age group were selected randomly for balanced age distribution in the subsample with size of 55 for further measuring. The subsample of 55 was selected to detect difference in serum micronutrients concentration between anemic and non-anemic group, assuming a two tailed test, with *α* = 0.05 and *β* = 0.20. These 5 age groups included the key growth periods and weaning time.

Singleton infants aged 0–18 months whose gestational age was 38–42 weeks at delivery and who were free of serious disease (including serious diseases of heart and brain, digestive system and urinary system, serious infective diseases) or congenital malformations were selected through a multistage sampling technique. We excluded infants who experienced diarrhea or upper respiratory tract infections in the last month or received antibiotics or micronutrient supplement therapy in the last 2 months. A selection of primary sampling units (28 villages in 2 counties) was based on a probability proportional to population size, and we randomly selected 12 households with 0–18 month-old singleton infants from each village. In all, 336 infants were enrolled in the study between March and October of 2010 and a stratified subsample of 55 infants was selected from all 336 infants (n = 11/group) for further laboratory studies.

### Ethical considerations

The study protocol and informed consent procedure were approved by the Medical Ethic Review Committee of Xi’an Jiaotong University (Number: 2010009). Informed consent was obtained from each infant’s primary caregiver.

### Data collection and blood sampling

Well-trained researchers used a structured questionnaire to collect information about household socio-demographic characteristics and infant feeding practices during in-home interviews. Infant’s birth date was obtained according to the immunization card presented during the interview.

Infant weight and height were measured using standardized methods described by the World Health Organization (WHO, 1995). Weight was measured to the nearest 10 g on an electronic scale. Recumbent length was measured to the nearest mm on a length board [11].

Weight-for-age Z-score (WAZ), height-for-age Z-score (HAZ), and weight-for-length Z-score (WHZ) was calculated with 2006 WHO Anthro and WHO Child Growth Standards (WHO, 2006). Malnutrition including underweight, stunting and wasting were defined from WAZ, HAZ, and WHZ, respectively, as <2 standard deviations than the mean [12].

Three consecutive days of food records, were used to assess the infant dietary intake. Complementary foods were recorded and calculated, and the amount of each complementary food was divided into its ingredients. The total nutrient intake was calculated from the sum of the amount of foods consumed multiplied by the nutrient content. For complementary foods, nutrient content was derived from the electronic version of the 2002 China Food Composition Tables or food ingredient labels.

We performed venipuncture in a peripheral vein and collected 2-mL blood samples among 55 infants of subsample. Every blood sample was divided into 2 tubes; one anticoagulated for whole blood and the other was left to clot and centrifuged to have serum for measurement of vitamin A (retinol), iron and zinc. To avoid photo-oxidation of vitamin A (retinol), tubes were wrapped in aluminum foil and were placed in a box away from light. For anticoagulated tubes, blood samples were labeled and analyzed for hematocrit and hemoglobin. Full blood counts were performed with an automated cell counter (F820, Hitachi Inc., Tokyo, Japan). For the other tubes, the serum were stored and frozen until analysis for vitamin A (retinol), iron, and zinc. Retinol concentration was measured in serum and the experimentation was done in faint light. It was measured by microfluorometric determination (RF-1501, Shimadzu Inc., Tokyo, Japan). Serum iron and zinc concentrations were measured by atomic absorption spectrophotometry (Z-8000, Hitachi Inc., Tokyo, Japan). Only 1-mL blood samples were collected in anticoagulated tubes among the rest 281 infants (except 55 subsamples from 336 infants), which were just analyzed for hematocrit and hemoglobin.

Anemia was defined as the WHO standard for infants aged 6–18 months [13] and the Chinese standard for infants aged 0–5 months [14] (Hb level is below 145 g/L for infants aged 0–29 days, below 90 g/L for infants aged 30–119 days, below 100 g/L for infants aged 120–179 days, and below 110 g/L for infants aged 180 days to 18 months). Anemia included mild, moderate and severe anemia and the definitions of them by age were as follows. Mild anemia: Hb level is between 90–144 g/L for infants aged 0–29 days, between 90–99 g/L for infants aged 120–179 days, between 90–109 g/L for infants aged 180 days to 18 months; Moderate anemia: Hb level is between 60–89 g/L for all age group infants; Severe anemia: Hb level is between 30–59 g/L for all age group infants.

Breast-feeding was defined including exclusive breast-feeding and predominant breast-feeding during the first 4 months of life. Exclusive breast-feeding: The infant had received only breast milk from his/her mother or a wet nurse, or expressed breast milk, and no other liquids or solids, with the exception of drops or syrups consisting of vitamins, mineral supplements or medicines. Predominant breast-feeding: The infant’s predominant source of nourishment had been breast milk. However, the infant might also have received water and water-based drinks (sweetened and flavored water, teas, infusions etc.); fruit juice; oral rehydration salts solution; drop and syrup forms of vitamins, minerals and medicines; and ritual fluids (in limited quantities). With the exception of fruit juice and sugar-water, no food-based fluid was allowed under this definition.

Low income family was defined as monthly family income less than 800 yuan.

### Statistical analyses

All analyses were conducted with SPSS 13.0 for Windows statistical package (SPSS Inc., Chicago, IL, USA). All socio-demographic and feeding behavioral variables were categorized before analysis. Wilcoxon rank sum tests were used to test differences of dietary intake and serum concentrations of iron, zinc, and vitamin A between anemic and non-anemic infants. χ^2^ tests were used to examine relationships among malnutrition, socio-demographic characteristics, feeding patterns, and anemia. Odds ratios (ORs) and 95% confidence intervals (CIs) were estimated with logistic regression modeling using the non-anemic group as the reference. *P*-values in all analyses were two-sided, and *P* < 0.05 was considered statistically significant.

## Results

### Prevalence of anemia in infants ≤18 months old

**Figure 1:**
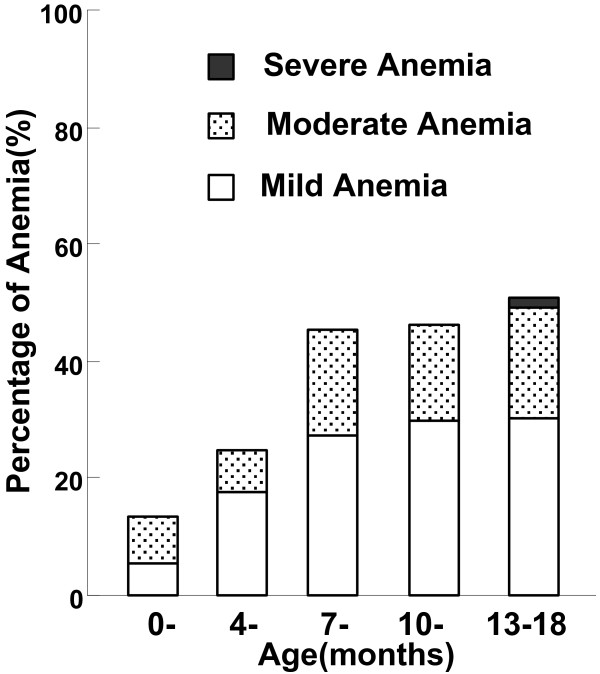
Percentage of anemia^* ^in 0–18 month-old infants by age group in rural Shaanxi province, China, 2010. ^*^Anemia: Hb level is below 145 g/L for infants aged 0–29 days, below 90 g/L for infants aged 30–119 days, below 100 g/L for infants aged 120–179 days, and below 110 g/L for infants aged 180 days to 18 months. Mild anemia: Hb level is between 90–144 g/L for infants aged 0–29 days, between 90–99 g/L for infants aged 120–179 days, between 90–109 g/L for infants aged 180 days to 18 months; Moderate anemia: Hb level is between 60–89 g/L for all age group infants; Severe anemia: Hb level is between 30–59 g/L for all age group infants.

Among 336 included infants, 118 (35.12%) had anemia, but there was no significant sex difference (39.11% male *vs*. 30.57% female, *P* > 0.05). As shown in Figure 1, anemia prevalence increased with age, from 13.33% (10/75) among infants aged 0–4 months to 50.82% (33/65) among them aged 13–18 months (*P* < 0.001).

The prevalence rates of mild, moderate, and severe anemia were 21.73% (73/336), 13.10% (44/336), and 0.30% (1/336), respectively. Mild and moderate anemia both increased with age and peaked at 13–18 months of age at prevalence rates of 30.77% (20/65) and 18.46% (12/65), respectively. In the entire sample, only one severely anemic infant was found in the 13–18 month group. The total anemic group was comprised of mild, moderate, and severe anemia at percentages of 61.86% (73/118), 37.29% (44/118), and 0.85% (1/118) respectively.

### Prevalence of malnutrition

**Figure 2:**
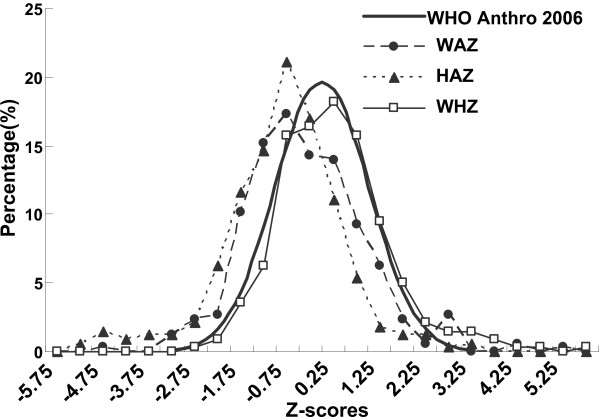
Height for age, Weight for age and Weight for height Z-scores^* ^for 0–18 month-old infants in rural Shaanxi province, China, 2010. ^*^Weight-for-age Z-score (WAZ), height-for-age Z-score (HAZ), and weight-for-length Z-score (WHZ) was calculated with 2006 WHO Anthro and WHO Child Growth Standards [WHO, 2006].

**Figure 3:**
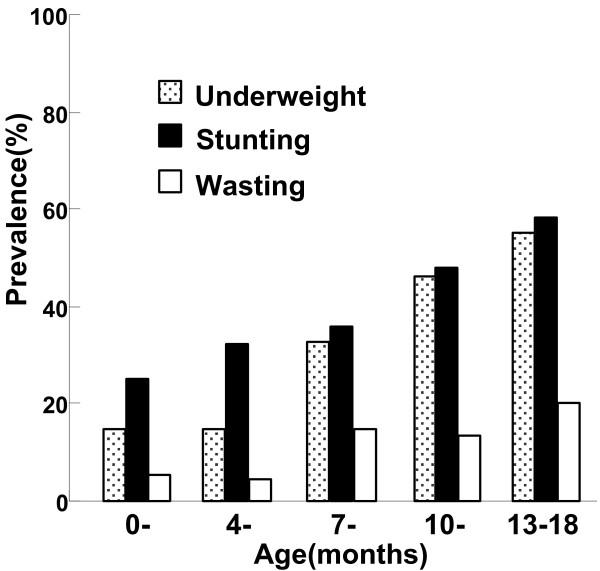
Prevalence of malnutrition^* ^among 0–18 month-old infants in rural Shaanxi province, China, 2010. ^*^Malnutrition includes underweigtht, stunting and wasting. Underweight: WAZ < 2 standard deviations than the mean; Stunting: HAZ < 2 standard deviations than the mean; Wasting: WHZ < 2 standard deviations than the mean [WHO, 2006].

Compared with the WHO reference values, the distributions of WAZ and HAZ were significantly shifted to the left (Figure 2). The prevalence of malnutrition among included infants was 32.14% (108/336) for underweight, 39.58% (133/336) for stunting, and 11.31% (38/336) for wasting. As shown in Figure 3, the prevalence rate of underweight or stunting increased with age.

### Anemia and associated socio-demographic and feeding practice risk factors

The prevalence of anemia in rural infants was associated with birth weight, sibling number, mother’s education, family income, persons per room, and feeding habits. Compared with their normal birth weight peers, infants with lower birth weight were at increased risk of anemia (OR 4.85; 95%CI 1.12-20.96). When stratified by sibling number, this association was significant for those who had 2 or more siblings (OR 2.76; 95%CI 1.01-7.55), but not for those who had only one sibling (OR 0.98; 95%CI 0.60-1.59). Infants whose mothers completed high school (more than 9 years) had a lower prevalence of anemia (OR 0.36; 95%CI 0.13-0.99) compared with those that completed 3 years of education, but the difference was not significant when comparing those who had >9 and < 9 years education. Family income was also associated with the prevalence of anemia. Low family income was associated with an increased risk of anemia (OR 1.60; 95%CI 1.02-2.52). Compared with households containing <1.5 persons per room, there was an increased risk of anemia for infants living in households with >3 persons per room (OR 2.44; 95%CI 1.26-4.75) (Table 1).

**Table 1:** Socio-demographic characteristics and feeding practices of 0–18 month-old infants by anemia status in rural Shaanxi province, China, 2010

**Variable**	**Anemic**	**Non-anemic**	**Odds ratio (95% *CI*)**
Birth weight (n = 227)			
<2500 g	5	3	4.85 (1.12-20.96)
≥2500 g	56	163	1.00
Number of siblings (n = 335)			
≥2	10	7	2.76 (1.01-7.55)
1	38	75	0.98 (0.60-1.59)
0	70	135	1.00
Mother’s education (years) (n = 335)			
>9	14	33	0.36 (0.13-0.99)
≤9	65	127	0.43 (0.18-1.02)
≤6	26	46	0.48 (0.19-1.22)
≤3	13	11	1.00
Average income per month RMB (n = 336)			
≤800	58	82	1.60 (1.02-2.52)
>800	60	136	1.00
Person to room ratio (n = 336)			
>3	40	50	2.44(1.26-4.75)
≤3	59	110	1.64(0.89-3.00)
≤1.5	19	58	1.00
Breast-feeding^*^ (n = 336)			
No	32	27	2.63 (1.49-4.66)
Yes	86	191	1.00
Age of introduction of complementary food (n = 336)			
<4 months or >6 months	47	53	2.06 (1.27-3.34)
4-6 months	71	165	1.00
Frequency of high-quality complementary foods (n = 336)			
≥2 times per month	35	121	0.34 (0.21-0.55)
<2 times per month	83	97	1.00

^*^Breast-feeding: exclusive breast-feeding and predominant breast-feeding during the first 4 months of life.

Feeding practice also affected the prevalence of anemia. We determined that 82.44% of infants were breast-fed during the first 4 months of life, and 31.05% of them had anemia compared with 54.24% among those who were not breast-fed (*P* < 0.01). Non-breast-feeding during the first 4 months of life was associated with an increased risk of anemia (OR 2.63; 95%CI 1.49-4.66). The frequency and quality of complementary foods also played a role. Compared with infants who received complementary foods between 4 and 6 months, introducing complementary foods too early or too late increased the risk of anemia (OR 2.06; 95%CI 1.27-3.34). However, high-frequency feeding (>2 times/month) of protein-rich complementary food, such as egg, meat, or fish, was associated with a decreased risk of anemia (OR 0.34; 95%CI 0.21-0.55) (Table 1).

### Correlates between anemia and malnutrition

Among 118 infants with anemia, the prevalence rates of underweight, stunting, and wasting were 45.30% (53/118), 47.46% (56/118), and 18.64% (22/118), respectively. These values were significantly higher than those for infants without anemia (25.11%, 35.21%, and 7.33%, *P* < 0.01). Anemia in rural infants aged 0–18 months was significantly associated with being underweight (OR 2.42, 95%CI 1.50-3.88), stunted (OR 1.65, 95%CI 1.05-2.61), and wasted (OR 2.89, 95%CI 1.45-5.76) (Table 2).

**Table 2:** Growth retardation in 0–18 month-old infants by anemia status in rural Shaanxi province, China, 2010

**Variable**	**Anemic**	**Non-anemic**	**Odds ratio**
	**(n = 118)**	**(n = 218)**	**(95% CI)**
Underweight			
Yes (n = 108)	53	55	2.42(1.50-3.88)
No (n = 228)	65	163	1.00
Stunting			
Yes (n = 133)	56	77	1.65(1.05-2.61)
No (n = 203)	62	141	1.00
Wasting			
Yes (n = 38)	22	16	2.89(1.45-5.76)
No (n = 298)	96	202	1.00

### Iron, zinc, and vitamin A deficiencies in serum contribute to anemia

As shown in Table 3, we observed that dietary intake levels of iron, zinc, and vitamin A were lower than the reference (*P* < 0.01), but there was no statistical difference between infants with and without anemia (*P =* 0.746, 0.299, and 0.227, respectively). The difference of absolute iron intake between anemic infants and non-anemic infants was not statistically significant. But after comparing the absolute iron intake to RDA [15] we found anemic infants had lower average relative iron intake (percentage of absolute iron intake to iron RDA) than non-anemic infants (48.2% *vs.* 63.6%, *P* < 0.05). Furthermore, we discovered that iron, zinc, and Retinol (vitamin A) deficiencies in serum were inconsistent with dietary intake; only serum Retinol (vitamin A) level was lower than normal. However serum concentrations of iron, zinc, and Retinol (vitamin A) in anemic infants were lower than in those without anemia (*P =* 0.014, 0.027, and <0.001, respectively).

**Table 3:** **Dietary intake (day**^**-1**^**) and serum concentrations of iron, zinc, and vitamin A in 0–18 month-old infants by anemia status in rural Shaanxi province, China, 2010**

**Variable**	**Anemic**	**Non-anemic**	***P*-value**
Dietary intake (n = 336)	(n = 118)	(n = 218)	
Iron (mg/day)	1.2 ± 1.0	1.3 ± 0.9	0.746
Zinc (mg/day)	1.8 ± 0.6	2.0 ± 0.5	0.299
Vitamin A (μg/day)	66.7 ± 24.5	75.3 ± 27.3	0.227
Serum concentration (n = 55)	(n = 21)	(n = 34)	
Iron (μg/L)	881.2 ± 449.9	1227.9 ± 566.4	0.014
Zinc (μg/L)	610.0 ± 107.8	679.4 ± 117.1	0.027
Retinol (μmol/L)	0.5 ± 0.1	0.8 ± 0.1	<0.001

## Discussion

Anemia and growth abnormalities including stunting and/or underweight in infants aged 0–18 months in rural Shaanxi were still a major public health problem. Some disadvantageous socio-demographic characteristics including poor household, increased sibling number, lower maternal education, crowded living conditions, and low birth weight were significantly associated with anemia. And inappropriate feeding practices were also significantly associated with anemia. Furthermore, there were strong associations between malnutrition (growth abnormalities) and anemia among infants in these rural areas. When we investigated nutritional factors resulting in this issue, we found a special status. That was iron, zinc, and vitamin A deficiencies in serum were not consistent with dietary intake.

China has the largest population in the world with more than 70% of the people living in rural areas [10]. More than 16 million babies are born in each year since 2000. Although economic and social situations have greatly improved in the past 30 years, health disparities among different regions continue to increase. Malnutrition and associated diseases in children are still very common problems, especially in the remote inland counties [16-19]. Shaanxi is a typical inland province in northwestern China. We found that malnutrition and anemia were still very common among 0–18 month-old infants there; the prevalence (47.6%) of anemia in rural infants aged 6–18 months was much higher than in western China (40%) [19]. This survey used the WHO standard of anemia combined with the Chinese standard for infant <6 months old, which had different cut-off values of hemoglobin for different ages rather a unified hemoglobin cut-off value for all infants [13,14]. Our results demonstrated that anemia in 0–18 month-old infants in rural northwestern China was much more prevalent than in New Zealand [20]. They also indicated that infants from these rural areas were more likely to develop symptoms of malnutrition (growth abnormalities), such as underweight and stunting. The latter is an indicator of chronic malnutrition and is a retardation of linear growth, as measured by total body weight or height, that is the product of a cumulative history of stressful episodes that cannot be compensated by catch-up growth during more favorable periods.

Childhood anemia occurred simultaneously with malnutrition in rural Shaanxi, and >60% showed only mild anemia. This indicated that anemia among these rural infants was potentially associated with malnutrition. Underweight, stunting, and wasting all were risk factors for suffering from anemia [21].

Physical development and nutrition status are affected by genetic and environmental factors, as well as the interaction between them [22]. Environmental factors, especially socio-economic status and feeding habits play leading roles in determining physical development and nutritional status in the early years of life. Risk factors of anemia have been described in a number of studies [23-25]. In this population, infants who were born with low birth weight and from lower income family were more likely to suffer from anemia. It had been reported in disadvantaged families from Montreal [26] that low birth weight was associated with iron deficiency anemia. The multivariate analysis showed the effect of crowded living conditions and more siblings on the prevalence of anemia. The effect had been previously noted by others [27,28]. Lack of exclusive or predominant breast-feeding during the first 4 months of life was found to be associated with infant anemia in our study. Prolonged exclusively breasted feeding, such as over 6 months old, was reported to be predictor of infant anemia in developing countries [29]. The time of starting and the frequency and quality of complementary foods were significantly associated with anemia in this group of infants. So information of introducing proper complementary foods, such as iron-fortified cereal, egg since 4–6 months old should be delivered to mothers and caregivers efficiently. We found less maternal education was one of the risk factors to infant anemia. This risk factor had been identified in rural China [28]. Given that this was a cross-sectional and retrospective study, we were unable to fully control for recall bias, especially for maternal feeding behavior information. A larger prospective study is required to clarify the causal relationships among these factors with anemia in young children in rural northwestern China.

Accumulating evidence demonstrates that anemia is a common clinical manifestation of micronutrient deficiency, particularly iron, zinc, and vitamin A [21]. In this study, we investigated the dietary intake levels among 336 infants and serum concentrations of iron, zinc, and retinol (vitamin A) in a subset of 55 infants. Unlike previously published results [21], We found that lower levels of serum iron, zinc and retinol (vitamin A) concentrations were found among anemic infants. But we didn’t find any differences of dietary intake between anemic and non-anemic infants accordingly. This inconsistency might be due to following explanations. Diet intake was just one determinant factor of nutrient status. The levels of nutrients in blood could be affected by bioavailability or other factors such as genetic influences and physical activity. And some infants were nursing breast milk and complementary foods at the same time. Mineral level of breast milk usually varied greatly between different mothers. On the other hand, these results indicated that other important factors might be involved in serum micronutrient deficiencies [30,31]. For example, micronutrient absorption and immune system function might result in serum micronutrient deficiencies. In future studies, we will analyze the status of intestinal microflora and bioactive molecules in serum to attempt to elucidate the causes. Limitations of the present study include: (1) Use of dietary record method for assessment of dietary intake. This method has the advantage of being non-dependant on caregiver’s memory. However, three consecutive days of intake might not be well representative of long term dietary intake of infants. (2) Measuring serum iron alone is not a sensitive indicator for iron status among infants. This was done for financial issues of the study.

## Conclusions

In conclusion, this study demonstrated that disadvantageous socio-demographic characteristics and feeding patterns were highly associated with anemia in 0–18 month-old infants in rural areas of Shaanxi province in northwestern China. Health education programs that focus on breast-feeding and complementary feeding practices could be critical and practical strategies for preventing anemia and malnutrition in young children. These findings suggest the urgent need for a more effective infant nutritional policy and a comprehensive program that includes maternal and child health care delivery and nutrition education, as well as the necessity to pay more attention to improving the health of these infants that have already been affected.

## Competing interests

The authors declare that they have no competing interests.

## Authors’ contributions

WY was the principal investigator, designed the study, analyzed and interpreted the data and prepared the manuscript. XL contributed to the study design, helped to analyze and interpret the data and draft the manuscript. YL and SZ participated in its design and coordination of field work and data collection and helped to draft the manuscript. LL helped to analyze and interpreted the data and draft the paper. XW and WL took the responsibility for experiment. All authors read and approved the final manuscript.

## Pre-publication history

The pre-publication history for this paper can be accessed here:

http://www.biomedcentral.com/1471-2458/12/1127/prepub

## References

[B1] Pelletier DL The relationship between child anthropometry and mortality in developing countries: implications for policy, programs and future research J Nutr 1994 124 10 Suppl 2047S 2081S 7931716 10.1093/jn/124.suppl_10.2047S

[B2] Pelletier DL Frongillo EA Jr Habicht JP Epidemiologic evidence for a potentiating effect of malnutrition on child mortality Am J Public Health 1993 83 8 1130 1133 10.2105/AJPH.83.8.1130 8342721 PMC1695164

[B3] Truswell AS ABC of Nutrition: Malnutrition in the Third World-1 Brit Medical J (Clin Res Ed) 1985 191 525 528 10.1136/bmj.291.6494.525 PMC1416532 3928036

[B4] WHO The World Health Report 2005: Make every mother and child count 2005 Geneva: WHO Press 10.1080/14034940500217037 16332605

[B5] UNICEF The state of the World’s children 1999 New York: Oxford University Press

[B6] Chen Y Tang S Le H Yu X Wang D Hao M Compare Health development of rural areas in different districts of China: About social economy and inhabitant’s health condition Chinese Health Economic 2006 25 2 37 38

[B7] Wang YP Miao L Dai L Zhou GX He CH Li XH Li Q Li MR Zhu J Liang J Mortality rate for children under 5 years of age in China from 1996 to 2006 Public Health 2011 125 301 307 10.1016/j.puhe.2011.01.003 21524772

[B8] Wang YP Miao L Qian YQ Liang J Wu YQ Zhu J Analysis of under 5 years old children mortality and the leading death cause in China from 1996 to 2000 Zhonghua Yu Fang Yi Xue Za Zhi 2005 39 4 260 264 16194383

[B9] Chen CM He W Fu ZY Wang Y Fu G Chang SY Analysis on the changes of nutritional situation in past 15 years (1990–2005) in China the 15 - year establishment of Chinese food and nutrition surveillance system( CFNSS) Wei Sheng Yan Jiu 2006 35 6 762 764,774 17290761

[B10] Wang X Wang Y Kang C Feeding practices in 105 counties of rural China Child Care Health Dev 2005 31 4 417 423 10.1111/j.1365-2214.2005.00523.x 15948878

[B11] WHO Physical status: the use and interpretation of anthropometry: report of a WHO expert committee. Technical report series, No 854 1995 854 Geneva: WHO Press 8594834

[B12] WHO Child growth standards: Length/height-for-age, Weight-for-age, Weight-for-height 2006 Available at: http://www.who.int/childgrowth/standards/height%20for%20age/en/index.html, http://www.who.int/childgrowth/standards/weight%20for%20age/en/index.html, http://www.who.int/childgrowth/standards/weight%20for%20height/en/index.html

[B13] WHO/UNICEF/UNU Iron Deficiency Anemia Assessment, Prevention and Control: A guide for programme managers 2001 Geneva: WHO (WHO/NHD/01.3) http://www.who.int/nut/documents/ida_assessment_prevention_control.pdf

[B14] Zheng J Wu H Diagnosis and treatment of child nutritional anemia Chinese Community Doctors 2010 12 2 3 5

[B15] Society CN Chinese dietary reference intakes 2000 Beijing, China: China Light Industry Publishing House

[B16] Chang Y Xian ZD He W Chang SY Ma HJ Chen CM Child malnutrition in China–present status and changing trend Biomed Environ Sci 1996 9 2–3 164 180 8886327

[B17] Popkin BM Keyou G Zhai F Guo X Ma H Zohoori N The nutrition transition in China: a cross-sectional analysis Eur J Clin Nutr 1993 47 5 333 346 8319669

[B18] Zeng L Yan H Guo X Dang S Xie H Analysis on malnutrition of children under 3 years old in 40 poor counties in the western areas of China Chinese Journal of Public Health 2003 19 1 55 58

[B19] Zeng L Yan H Chen Z Dang S Xie H Analysis on the prevalence of anemia among children under 3-year-old in 5 provinces in Western China Chinese Journal of Epidemiology 2004 25 3 225 228 15200935

[B20] Grant CC Wall CR Brunt D Crengle S Scragg R Population prevalence and risk factors for iron deficiency in Auckland, New Zealand J Paediatr Child Health 2007 43 7–8 532 538 17635681 10.1111/j.1440-1754.2007.01129.x

[B21] Lynch S Stoltzfus R Rawat R Critical review of strategies to prevent and control iron deficiency in children Food Nutr Bull 2007 28 4 Suppl S610 S620 18297898 10.1177/15648265070284S413

[B22] Tanner JM Population differences in body size, shape and growth rate. A 1976 view Arch Dis Child 1976 51 1 1 2 10.1136/adc.51.1.1 942224 PMC1545872

[B23] Saito K Korzenik JR Jekel JF Bhattacharji S A case–control study of maternal knowledge of malnutrition and health-care-seeking attitudes in rural South India Yale J Biol Med 1997 70 2 149 160 9493847 PMC2589065

[B24] de Waal HA D-v Environmental factors influencing growth and pubertal development. Environ Health Perspect 1993 101 Suppl 2 39 44 10.1289/ehp.93101s239 PMC1519930 8243404

[B25] Miller JE Korenman S Poverty and children’s nutritional status in the United States Am J Epidemiol 1994 140 3 233 243 8030626 10.1093/oxfordjournals.aje.a117242

[B26] Lehmann F Gray-Donald K Mongeon M Di Tommaso S Iron deficiency anemia in 1-year-old children of disadvantaged families in Montreal Can Med Assoc J 1992 146 9 1571 1577 1571868 PMC1488517

[B27] Hadler MC Juliano Y Sigulem DM [Anemia in infancy: etiology and prevalence] J Pediatr (Rio J) 2002 78 4 321 326 14647764

[B28] Ma Y Bi Y Yan H Deng L Liang W Wang B Zhang X The application of decision tree in the research of anemia among rural children under 3-year-old Zhonghua Yu Fang Yi Xue Za Zhi 2009 43 5 434 437 19535001

[B29] Meinzen-Derr JK Guerrero ML Altaye M Ortega-Gallegos H Ruiz-Palacios GM Morrow AL Risk of infant anemia is associated with exclusive breast-feeding and maternal anemia in a Mexican cohort J Nutr 2006 136 2 452 458 16424127 10.1093/jn/136.2.452

[B30] Chandra RK Nutrition and the immune system from birth to old age Eu.r J. Clin. Nutr 2002 56 Suppl 3 S73 S76 10.1038/sj.ejcn.1601492 12142969

[B31] Cunningham-Rundles S McNeeley DF Moon A Mechanisms of nutrient modulation of the immune response J Allergy Clin Immunol 2005 115 6 1119 1128 quiz 1129 10.1016/j.jaci.2005.04.036 15940121

